# Vitamin D prescribing practices among clinical practitioners during the COVID‐19 pandemic

**DOI:** 10.1002/hsr2.691

**Published:** 2022-07-11

**Authors:** Edward B. Jude, Nikolaos Tentolouris, Ashu Rastogi, Moi H. Yap, Hermelinda C. Pedrosa, Stephanie F. Ling

**Affiliations:** ^1^ Department of Diabetes and Endocrinology Tameside and Glossop Integrated Care NHS Foundation Trust Ashton‐under‐Lyne UK; ^2^ Department of Diabetes and Endocrinology The University of Manchester Manchester UK; ^3^ Department of Diabetes and Endocrinology Manchester Metropolitan University Manchester UK; ^4^ 1st Department of Internal Medicine, Medical School, National and Kapodistrian University of Athens Laiko General Hospital Athens Greece; ^5^ Department of Diabetes and Endocrinology Post Graduate Institute of Medical Education and Research Chandigarh India; ^6^ Department of Diabetes and Endocrinology, Endocrinology Unit, Research Centre, Taguatinga Regional Hospital Secretariat of Health Brasilia‐DF Brazil

**Keywords:** COVID‐19, endocrinology and metabolic disorders, vitamin D

## Abstract

**Background and Aims:**

COVID‐19 has caused devastation globally. Low vitamin D status, particularly during the winter months, remains commonplace around the world, and it is thought to be one of the contributing factors toward causation and severity of COVID‐19. Many guidelines do not recommend vitamin D for the treatment or prevention of the disease. Hence, we set out to conduct a global survey to understand the use and prescribing habits of vitamin D among clinicians for COVID‐19.

**Methods:**

An online anonymous questionnaire was sent to clinicians enquiring about their prescribing habits of vitamin D and personal use of vitamin D. Data of the survey were collected between January 15, 2021, and February 13, 2021.

**Results:**

Four thousand four hundred forty practicing clinicians were included in the analysis, with the majority of those responding from Asia, followed by Europe. 82.9% prescribed vitamin D before COVID‐19, more commonly among general practitioners (GPs) in comparison with medical specialists, and Asian clinicians were more likely to prescribe vitamin D in comparison with Caucasian physicians (*p* < 0.01). GPs were also more likely to prescribe vitamin D prophylactically to prevent COVID‐19 in comparison with medical specialists (OR 1.47, *p* < 0.01). Most GPs (72.8%) would also prescribe vitamin D to treat COVID‐19 in comparison with medical specialists (OR 1.81, *p* < 0.01), as well as more Asian in comparison with Caucasian physicians (OR 4.57, *p* < 0.01). 80.4% of respondents were taking vitamin D, more so in the 45–54 and 65–74 age groups in comparison with the 18–24 years category (OR 2.15 and 2.40, respectively, both *p* < 0.05), many of whom did so before COVID‐19 (72.1%).

**Conclusion:**

This survey has shown that many clinicians would prescribe vitamin D for the prevention and treatment of COVID‐19. The majority would also recommend measuring vitamin D levels, but not so in patients with COVID‐19.

## 1 INTRODUCTION

The infection caused by the severe acute respiratory syndrome coronavirus 2 (SARS‐CoV‐2) and its associated severe acute respiratory syndrome (SARS),^1^ named by the World Health Organization (WHO) as coronavirus disease 2019 (COVID‐19) in January 2020,^2^ has unequivocally become the greatest healthcare challenge faced by humankind in the 21st century. Following its incredibly rapid and severe spread, a global pandemic was declared by the WHO on March 11, 2020.^3^ At that time, the number of affected individuals outside its originating country of China increased 13‐fold, the pandemic spread in 114 countries, and 118,000 cases were registered, sadly causing nearly 4300 deaths. As of now, over 1 billion people worldwide have contracted COVID‐19 and over 6 million deaths have occurred due to infection with SARS‐CoV‐2.

Several factors have been studied as possibly affecting the transmission and outcomes of COVID‐19, including age, ethnicity, metabolic diseases, as well as environmental and social parameters such as temperature and air pollution.^4^, ^5^, ^6^, ^7^, ^8^ Interestingly, some of these factors have also been linked to higher risk of vitamin D deficiency,^9^ while a large number of studies regarding the possible association between vitamin D levels and the risk of COVID‐19 contraction and worse outcomes have been published.^10^, ^11^, ^12^, ^13^, ^14^, ^15^ This link between vitamin D levels and COVID‐19 outcomes can be explained based on the immunomodulatory actions of calcitriol, the interaction with the renin‐angiotensin‐aldosterone system (RAAS), as well as its protection against endothelial dysfunction and thrombosis.^16^, ^17^, ^18^ Moreover, vitamin D suppresses cytokine production by simultaneously boosting the innate immune system, thus reducing the SARS‐CoV‐2 load, and decreasing the overactivation of the adaptive immune system to immediately respond to the viral load.^19^

At the beginning of the pandemic when effective drugs and vaccines were not available,^20^, ^21^ there were physicians who prescribed treatments that were not proven to be useful: chloroquine, hydroxychloroquine (HCQ), azithromycin,^22^, ^23^ ivermectine,^24^ and nitroxamide,^25^ and advocate their use either for prevention or early treatment based on medical ethical autonomy to prescribe. The *Solidarity Therapeutics* Trial,^26^ a very large randomized clinical trial (RCT) held in 30 countries over a period of 6 months, has shown little or no effect for remdesivir, HCQ, lopinavir/ritonavir, and interferon regimens on 28‐day mortality or in‐hospital course among patients hospitalized with COVID‐19. Immunomodulatory agents (convalescent plasma, intravenous infusion of immunoglobulin, eculizumab) have also not shown any conclusive effects.^20^

Previous data from a meta‐analysis of 25 RCTs showed that vitamin D3 2000 IU daily supplementation has a protective action against acute respiratory infections and prophylactic use for COVID‐19 has gained attention.^27^ There has been much controversy toward its use and even more concerning the adequate dose.^28^ Despite the lack of clinically robust data to support this therapy, many physicians eagerly prescribe generous doses of vitamin D, not in accordance with guidelines. A Brazilian study^29^ using a high single oral dose of 5000 μg (200,000 IU) did not show any benefit on hospital stay duration in individuals with severe COVID‐19 (*n* = 240); however, it should be mentioned that vitamin D supplementation began after the onset of moderate to severe COVID‐19. Moreover, a smaller Indian trial involving 40 mild or asymptomatic patients with vitamin D deficiency, showed that a greater proportion of patients with SARS‐CoV‐2 infection turned SARS‐CoV‐2 RNA negative after 21 days of vitamin D supplementation.^30^ A recent study that evaluated the impact of vitamin D supplementation on the outcomes of COVID‐19 found that supplementation was associated with reduced hospital stay and mortality and suggested the regulation of several factors such as the inducible nitric oxide synthase, interleukins, and cathelicidin‐LL3.^31^ In addition, a UK‐based multicentre retrospective observational study (*n* = 986) showed that high‐dose cholecalciferol booster therapy (approximately ≥280,000 IU in a time period of up to 7 weeks before hospital admission as part of routine clinical practice in cases of vitamin D insufficiency or deficiency) reduced risk of COVID‐19 mortality regardless of baseline serum 25(OH)D levels.^32^ A recent meta‐analysis found that vitamin D supplementation reduces admissions to intensive care units, but not mortality, in patients with severe COVID‐19.^9^

However, in the United Kingdom, the National Institute for Health and Care Excellence (NICE), Public Health England, and the Scientific Advisory Committee on Nutrition rapid guideline admit the lack of evidence, emphasizing the need for more research and reinforcing the previous UK government seasonal (October to March) policy for a daily 400 IU for both adults and children, and throughout the year for ethnic minority groups.^33^

Despite the guidelines, there is a lot of support for the use of vitamin D in COVID‐19.^34^, ^35^, ^36^ Therefore, to understand better the role of clinicians in the use of vitamin D, we conducted an international web‐based survey of practicing clinicians. This global survey is the first of its kind and was done to better understand clinicians' views on vitamin D supplementation during the ongoing SARS‐CoV‐2 pandemic.

## 2 METHODS

A survey of the use of cholecalciferol by clinicians and its association with COVID‐19 was conducted. The inclusion criterion was people who are practicing clinicians. To reach participants worldwide, this survey was conducted through an online questionnaire‐based study, hosted via Google Forms. The survey was anonymized, and no identifiable personal data were collected during the process. Survey data were collected between January 15, 2021, and February 13, 2021.

The questionnaire consisted of five sections with a total of 24 questions. The first section concerned demographic information, including specialty, ethnicity, age, country in which the respondent was practicing, and the respondent's involvement in COVID‐19 management and treatment. The second section referred to information regarding the prescription of cholecalciferol to prevent and treat COVID‐19. The third section concerned the personal consumption of cholecalciferol as a supplement, before and after the start of the COVID‐19 pandemic. The fourth section concerned any comorbidities of the respondent. The final section asked about opinions on the prescription of cholecalciferol in people with vitamin D deficiency. As our targeted volunteers were clinicians, we advertised our survey on Medscape^37^ and through clinicians' networks, including the Association of British Clinical Diabetologists in the United Kingdom, and locally in other countries. A full copy of the questionnaire is included in the Supporting Information: S1 File. Measured variables were the responses by the participating clinicians. Since this study was an anonymized survey and no personal information was obtained or stored from participants, it was deemed not necessary to require ethical approval and was given approval by the local research committee.

### 2.1 Statistical methods

Data are expressed as *n* (%). Unadjusted logistic regression was used to determine whether participants were more or less likely to respond in a certain manner to each survey question, based on the following characteristics: specialty, ethnicity, age group, and whether the respondent cared for patients with COVID‐19. The a priori levels of significance were two‐sided and *p* < 0.05 were considered statistically significant. All analyses were carried out in Stata v 14.0 (StataCorp).

## 3 RESULTS

A total of 5118 healthcare professionals responded; we excluded 678 responders because they were not practicing clinicians. The remaining 4440 practicing clinicians were included in the analysis. The demographics of the study participants are shown in Table 1.

**Table 1:** Demographic characteristics of the participants

Demographic characteristics	*n* (%)
Ethnicity	
Caucasian	1062 (23.9)
Asian (including Middle East, South and East Asian)	3221 (72.6)
Other (including Black, Latin, and mixed race)	150 (3.4)
Not stated	7 (0.1)
Total	4440 (100)
Location of responder	
Asian	3048 (68.7)
India	2993
All other Asian countries	55
Europe	936 (21.1)
America (North, Central, and South)	434 (9.8)
Africa	16 (0.3)
Not stated	6 (0.1)
Respondents managing patients with COVID‐19	3172 (71.4)
Age group	
18–24	39 (0.9)
25–34	365 (8.2)
35–44	1255 (28.2)
45–54	1579 (35.6)
55–64	882 (19.9)
65–74	276 (6.2)
Specialty	
Medicine (all specialties in hospitals)	2292 (51.6)
General practice/family medicine	1647 (37.1)
Surgery (all specialists in hospitals)	244 (5.5)
Other	257 (5.8)
Setting of contact with COVID‐19 patients	
Primary care	609 (13.7)
Hospital‐in patients: nonintensive care unit	1357 (30.6)
Intensive care unit	504 (11.3)

Most participants were from Asia and mainly from India, followed by Europe. Most of the participants practiced medicine as specialists or general practice (GP)/family physicians and almost 64% were in the age group of 35–54 years. Most respondents (71.4%) participated in the care of patients with COVID‐19, and most often they managed patients in an outpatient setting.

Regarding vitamin D prescribing patterns by participating clinicians before the pandemic, 4389 respondents answered this question, of whom a total of 3639 respondents (82.9%) prescribed vitamin D before the start of the COVID‐19 pandemic. Compared to medical specialists, general/family practitioners were significantly more likely to prescribe vitamin D (odds ratio [OR] 1.36 [95% confidence interval {CI} 1.13–1.64]; *p* < 0.01). Asian clinicians were more likely to prescribe vitamin D in comparison with Caucasian physicians (OR 2.98 [2.52–3.53]; *p* < 0.01), as were physicians involved versus those not involved in the care of patients with COVID‐19 (OR 2.27 [1.93–2.67]; *p* < 0.01). No significant relationship was found in prescribing patterns of vitamin D prepandemic between other ethnicities versus Caucasians. Other specialties and allied health professionals (AHPs) versus medicine specialists combined, were less likely to prescribe vitamin D (OR 0.19 [0.14–0.25]; *p* < 0.01). There were no significant relationships in prepandemic vitamin D prescribing patterns between age groups. These findings are summarized in Table 2.

**Table 2:** Vitamin D prescribing patterns of participants before the COVID‐19 pandemic (odds ratio, 95% confidence intervals)

	Odds ratio	95% CI
General/family practitioners versus medicine specialists	1.36	1.13–1.64*
Surgery versus medicine specialists	0.52	0.38–0.70*
Other specialties versus medicine specialists	0.19	0.14–0.25*
Asians versus Caucasians	2.98	2.52–3.53*
Other ethnicities versus Caucasians	1.09	0.74–1.59
Physicians managing patients with COVID‐19 (yes vs. no)	2.27	1.93–2.67*

* *p* < 0.01.

With regard to the question “Would you prescribe vitamin D to prevent COVID‐19?,” 4393 answered. A total of 3385 (77.1%) clinicians responded that they would prescribe vitamin D to prevent COVID‐19. General/family practitioners were significantly more likely to prescribe prophylactic vitamin D in comparison with medical specialists (OR 1.47 [1.25–1.72], *p* < 0.01); surgery was not significantly different from medicine (OR 1.16 [0.84–1.60], *p* = 0.35). Other specialties and AHPs combined were significantly less likely to prescribe vitamin D in comparison with medical specialists (OR 0.46 [0.35–0.6], *p* < 0.01). Both Asian (OR 3.66 [3.13–4.27], *p* < 0.01) and respondents from other ethnicities (OR 1.47 [1.02–2.11], *p* = 0.04) were more likely to prescribe prophylactic vitamin D for COVID‐19 when compared to Caucasian respondents. Respondents in the age category 25–34 years were less likely to prescribe prophylactic vitamin D in comparison with younger respondents in the 18–24 years age category (OR 0.35 [0.14–0.86], *p* = 0.02); there were no other significant differences by age category. Respondents who manage patients with COVID‐19 were more likely to prescribe prophylactic vitamin D (OR 2.73 [2.35–3.16], *p* < 0.01) when compared to those who do not manage patients with COVID‐19.

Regarding the question “Would you prescribe vitamin D to treat COVID‐19?,” all participants (4440) answered this question. A total of 3232 (72.8%) answered that they would prescribe vitamin D to treat active COVID‐19 patients. Compared to medical specialists, general/family practitioners were significantly more likely to prescribe vitamin D to treat COVID‐19 (OR 1.81, [95% CI 1.55–2.10], *p* < 0.01). Surgery was not significantly different from medicine (OR 1.33 [0.98–1.80], *p* = 0.06). Compared to Caucasian respondents, Asian respondents were significantly more likely to prescribe vitamin D to treat COVID‐19 (OR 4.57 [3.93–5.31], *p* < 0.01), as were respondents from other non‐Caucasian ethnicities (OR 1.57 [1.11–2.23], *p* = 0.01). Moreover, in comparison to respondents in the 18–24 years age category, those in the 25–34 years category (OR 0.30 [0.12–0.74], *p* = 0.01), the 55–64 years category (OR 0.41 [0.17–0.99], *p* = 0.04) and the 65–74 years category (OR 0.38 [0.15–0.93], *p* = 0.03) were significantly less likely to prescribe vitamin D to treat COVID‐19. No analysis was performed for respondents who manage patients with COVID‐19 versus those who do not, as the active clinical practice of a respondent was likely to bias prescribing decisions.

Concerning the question “Do you take Vitamin D yourself?,” 4067 respondents answered this question. A total of 3271 respondents (80.4%) answered that they take vitamin D themselves. Compared with medical specialists, general/family practitioners (OR 1.37, 95% CI 1.16–1.63, *p* < 0.01) and surgical specialists (OR 2.46 [1.58–3.83], *p* < 0.01) were more likely to be taking vitamin D themselves. Other specialties and AHPs combined were not significantly different compared to medical specialists (OR 0.95 [0.70–1.30], *p* = 0.74). Clinicians from Asian ethnic groups (OR 2.35 [1.99–2.77], *p* < 0.01) and respondents from other ethnicities (OR 2.11 [1.35–3.32], *p* < 0.01) were more likely to be taking vitamin D themselves when compared to Caucasian respondents. Compared with the 18–24 years category, those in the 45–54 years category (OR 2.15 [1.07–4.32], *p* = 0.03), the 65–74 years category (OR 2.40 [1.13–5.14], *p* = 0.02) and the ≥74 years category (OR 3.60 [0.31–11.43], *p* = 0.03) were more likely to be taking vitamin D themselves. There were no significant differences in the other age categories. In addition, respondents who manage COVID‐19 were more likely to be taking vitamin D themselves in comparison to those who do not (OR 1.69 [1.44–1.99], *p* < 0.01).

Regarding the question “When did you start taking vitamin D?,” 2997 respondents answered this question. A total of 2160 respondents (72.1%) said that they were already taking vitamin D before the start of the COVID‐19 pandemic.

With regard to the question “Were you vitamin D deficient before commencing treatment?” 2294 respondents answered this question who knew their vitamin D status. A total of 1600 respondents (69.8%) were vitamin D deficient before starting treatment. Asian respondents were significantly more likely to be vitamin D deficient (OR 1.57, 95% CI 1.27–1.95, *p* < 0.01) when compared to Caucasian respondents. Those from other non‐Caucasian ethnicities were not significantly different when compared to Caucasian respondents (OR 0.92 [0.59–1.45], *p* = 0.72). Compared with those in the 18–24 years age category, those in the 65–74 years category were less likely to be vitamin D deficient (OR 0.35 [0.12–0.98], *p* = 0.04). There were no other differences by age category.

All 4440 respondents answered the question regarding whether they would recommend vitamin D to a family member who had COVID‐19. 1698 respondents (38.2%) said that they would always recommend vitamin D to a family member, 2032 respondents (45.8%) said that they would recommend it only it their family member was deficient, and 230 respondents (5.2%) said that they would not recommend vitamin D at all, and the remainder of respondents said that they did not know. Compared to medical specialists, general/family practitioners (OR 2.30 [1.66–3.19], *p* < 0.01) and surgical specialists (OR 2.21 [1.07–4.56], *p* = 0.03) were more likely to recommend vitamin D to a family member. Furthermore, compared with Caucasian respondents, Asian respondents were more likely to recommend vitamin D to a family member (OR 3.10 [2.35–4.09], *p* < 0.01) and those who manage patients with COVID‐19 were also more likely to recommend vitamin D to a family member, compared to those who do not manage COVID‐19 (OR 1.44 [1.09–1.91], *p* = 0.01).

Regarding whether all patients should be tested for vitamin D deficiency, all 4440 respondents answered the question.  Two thousand four hundred ten respondents (54.3%) answered yes. Specialties apart from surgery and general/family practice combined with AHPs were more likely to recommend universal vitamin D testing compared to medical specialists (OR 1.39 [1.07–1.81], *p* = 0.01). Compared with Caucasian respondents, Asian respondents were more likely to agree with universal vitamin D testing (OR 1.20 [1.05–1.38], *p* = 0.01). Respondents who manage patients with COVID‐19 were more likely to say that all patients should be tested for vitamin D deficiency compared to those who do not manage COVID‐19 (OR 1.50 [1.32–1.7], *p* < 0.01).

Finally, with respect to the statement: “Patients with COVID‐19 need not be tested for vitamin D deficiency,” all 4440 respondents answered. Two thousand seven hundred forty‐six (61.9%) agreed that this is not necessary. Specialties apart from surgery and general/family practice combined with AHPs were less likely to agree (OR 0.43 [0.33–0.56], *p* < 0.01). Compared with Caucasian respondents, Asian respondents were more likely to agree that patients with COVID‐19 do not need to be tested for vitamin D deficiency (OR 1.66 [1.44–1.91], *p* < 0.01). Respondents who manage patients with COVID‐19 compared to those who do not were more likely to agree with the above statement (OR 1.80 [1.58–2.06], *p* < 0.01).

## 4 DISCUSSION

The present study was performed to assess the knowledge and practices regarding vitamin D prescription among healthcare practitioners (HCPs) from different geographical regions and different specialties across the globe during the COVID‐19 pandemic.

Vitamin D deficiency is highly prevalent globally, which has led to food fortification programs, especially in temperate countries with less sunshine. However, subtropical and tropical countries were not found to be free from vitamin D deficiency, despite plenty of sunshine. In addition, a low vitamin D level has been associated with depression.^38^ Depression is common among the general population during the pandemic^39^ and in COVID‐19 patients.^40^ There has been a renewed interest in vitamin D playing a therapeutic role during the COVID‐19 pandemic, considering its immunomodulatory properties affecting both the innate and cell‐mediated immune system, antioxidative effect, inhibition of ACE2 expression and pleiotropic effect on endothelial function.^15^, ^16^, ^17^, ^18^, ^41^, ^42^ Observational studies from Italy and Spain at the onset of pandemic suggested poor outcomes with COVID‐19 in people with vitamin D deficiency.^43^, ^44^ Thus, vitamin D usage has increased for respiratory infections, including SARS‐CoV‐2 infection.

We endeavored to better understand the prescribing practices of HCPs regarding the usage of vitamin D during the COVID‐19 pandemic. A total of 72.8% of respondents to this survey were of Asian ethnicity, and 63.9% of respondents belonged to age groups covering 35–54 years. It is very likely that younger age group HCPs are prioritized for the care of SARS‐CoV‐2 patients and were more willing to share their experiences regarding vitamin D in the present survey.

The shared experiences were mostly from managing COVID‐19 patients in an outpatient setting, rather than from respondents involved in critical care or in‐hospital care. Most of the HCPs prescribed vitamin D even before the COVID‐19 pandemic for varied reasons, with Asian HCPs almost three times more likely to prescribe vitamin D than their Caucasian counterparts. This observation could be due to heightened awareness of vitamin D deficiency in the Asian population and adverse outcomes from COVID‐19 in people with low vitamin D levels. Vitamin D deficiency is noticed irrespective of age, gender, socioeconomic class, or geography from either community or hospital‐based studies ranging from 40% to 100%, depending on various 25(OH)D cut‐offs.^45^, ^46^

The evidence for the role of vitamin D for the prevention of SARS‐CoV‐2 infection is scarce, though observational studies suggest that those who are vitamin D sufficient (25 (OH)D levels >20 ng/ml) or regularly on maintenance doses of cholecalciferol with pre‐COVID 25(OH)D are less likely to have respiratory tract infections or test positive for COVID‐19.^47^, ^48^, ^49^, ^50^ The present survey revealed that more than three‐quarters of respondents would like to prescribe vitamin D to prevent SARS‐CoV‐2 infection, and this finding was more frequent in general/family practitioners compared to hospital specialists.

HCPs of Asian ethnicity were more than three times more likely to suggest vitamin D therapy for prevention of COVID‐19 than Caucasians. This might suggest an amplified apprehension of adverse outcomes from SARS‐CoV‐2 infection that facilitated the pre‐emptive usage of therapies that are not backed by robust evidence, including vitamin D therapy. Similarly, most respondents said that they would prescribe vitamin D to treat patients with COVID‐19, with Asian respondents almost five times more likely than Caucasian respondents.

Interventional studies with vitamin D have used varying doses, for example, oral 200,000 IU of cholecalciferol as a one‐off dose,^29^ 400,000 IU as a bolus followed by 200,000 IU of cholecalciferol for two consecutive days (the first and second day of in‐hospital stay)^51^ and oral calcifediol 0.266 mg on Days 3 and 7 of admission, followed by weekly administration of the same dose until discharge,^52^ while others used various doses of cholecalciferol.^15^ The outcome of COVID‐19 related to risk of severe infections, hospitalization, intensive care unit stay, or mortality has, at best, provided inconsistent evidence from the available placebo‐controlled therapeutic cholecalciferol supplementation studies. The result of the present survey suggests that most respondents (72.8%) prefer to prescribe vitamin D for the treatment of COVID‐19, despite controversial evidence for the role of therapeutic vitamin D supplementation.

Most of the respondents opined that they would recommend vitamin D for their family members with COVID‐19 and only 5.2% were not of the opinion for therapeutic usage of vitamin D. Almost half of the respondents said that they would like to provide supplemental vitamin D in COVID‐19‐affected family members if found to be vitamin D deficient, and one third agreed to supplement vitamin D without a prior vitamin D level or irrespective of vitamin D levels. Also, respondents of Asian ethnicity and those who are involved in the care of COVID‐19 patients were more likely to provide vitamin D to the affected family member as compared to Caucasian respondents or those not involved in direct COVID‐19 patient care, respectively.

Most of the respondents (80.4%) were themselves taking some form of vitamin D supplementation, even before the onset of the COVID‐19 pandemic. The self‐administration of vitamin D among HCPs is not a surprise, considering the widespread vitamin D deficiency in Asian countries, especially India, and highlights an increased awareness among the respondents regarding vitamin D deficiency and the associated ill‐effects on musculoskeletal, immune, and metabolic functions. In fact, a recently published study that evaluated the trends of vitamin D status around the world found that the prevalence of vitamin D deficiency had decreased in India during a period of 8 years, which was attributed to increased awareness and hence, increased supplementation.^53^ HCPs > 45 years of age were two to three times more likely to take vitamin D themselves, as compared to the younger cohort, <25 years of age. This observation may be due to more experience of the beneficial effect of cholecalciferol supplementation on vitamin D deficiency, or assumption of vitamin D sufficiency among younger respondents.

However, only around half of the respondents agreed that vitamin D testing should be performed for all patients during the COVID‐19 pandemic, irrespective of their specialty. Asian HCPs were 1.2 times more likely to concur with the need for vitamin D testing among all individuals. Moreover, those HCPs who manage patients with COVID‐19 were 1.5 times more likely to suggest vitamin D testing for all individuals.

Almost two‐thirds of HCPs agreed that testing may not be required for all COVID‐19 patients. Asian HCPs were 1.7 times and HCPs who manage COVID‐19 patients were 1.8 times more likely to concur with the lack of need for vitamin D testing in COVID‐19 patients. A heightened awareness of immunomodulatory effects, poor outcomes in vitamin D deficiency, and reduced morbidity and mortality outcomes in those COVID‐19 patients with 25(OH)D > 30 ng/ml may have rendered an opinion among HCPs for the futility of vitamin D testing, and instead to advocate vitamin D supplementation, irrespective of vitamin D status. Another reason could be the unavailability or lack of the standardization of vitamin D assays at peripheral health centers treating COVID‐19 patients. Moreover, even if vitamin D levels are available, there is very limited data regarding the concentration of 25(OH)D levels that would provide optimal health benefits for COVID‐19‐affected patients. For example, a 25(OH)D level of more than 40 ng/ml is purported to be associated with the least risk of chronic diseases and all‐cause mortality, and has plausible benefits for the prevention and treatment of COVID‐19 infection.^47^, ^48^, ^54^, ^55^

This web‐based survey is the first of its kind to enquire about the prevailing knowledge and practices regarding the role of vitamin D and prescription patterns during the COVID‐19 pandemic. The strength of this survey is that it includes multi‐ethnic HCPs across different specialties and from varied geographical regions. The questionnaire was inclusive, easy to comprehend and designed after discussion among investigators from across the globe, although its validity/reliability was not tested. Certainly, this study has limitations. Although Asian representation constituted the majority of respondents, the survey captures the practices of HCPs regarding vitamin D across specialties and a wide range of countries. Moreover, very limited data are available from the African continent and the results cannot be generalized in all countries. Furthermore, no assessment was made regarding the dose of vitamin D HCPs suggest for the prevention or treatment of COVID‐19. Finally, the survey was only conducted in English and was not translated into other languages.

The management of the COVID‐19 pandemic requires the vigilant surveillance of its course,^56^ as well as the implementation of both pharmaceutical and non‐pharmaceutical measures, including vaccinations and social distancing policies.^57^, ^58^, ^59^ Despite the deleterious effects of this pandemic on healthcare systems and the urgent need for powerful health policies, it has been found that all countries have weaknesses in these areas,^60^, ^61^, ^62^ rendering new research and implementation of effective strategies necessary. In light of these recent developments, several regimens have been proposed as possible contributors against this pandemic, including the use of vitamin D supplementation. However, due to the lack of robust evidence from high‐quality RCTs and of unanimous published guidelines regarding vitamin D supplementation and COVID‐19 outcomes, the attitude of HCPs toward its use in clinical practice was unknown. According to this survey, HCPs across the globe in a wide range of settings have acknowledged the importance of the role of vitamin D. Although definite recommendations for the use of vitamin D in the management of COVID‐19 are missing, this survey has shown that despite this lack of recommendations, vitamin D is increasingly being used for both the prevention and the treatment of COVID‐19.

## AUTHOR CONTRIBUTIONS

**Edward B. Jude**: Conceptualization; data curation; formal analysis; investigation; methodology; project administration; supervision; validation; writing—original draft; writing—review and editing. **Nikolaos Tentolouris**: data curation; formal analysis; project administration; writing—review and editing. **Ashu Rastogi**: Conceptualization; project administration; supervision; writing—original draft; writing—review and editing. **Moi H. Yap**: Data curation; methodology; project administration; writing—original draft; writing—review and editing. **Hermelinda C. Pedrosa**: Project administration; writing—review and editing. **Stephanie F. Ling**: Conceptualization; data curation; formal analysis; investigation; methodology; project administration; writing—original draft; writing—review and editing. All authors have read and approved the final version of the manuscript. The corresponding author had full access to all the data in this study and takes complete responsibility for the integrity of the data and the accuracy of the data analysis.

## CONFLICT OF INTEREST

The authors declare no conflict of interest.

## TRANSPARENCY STATEMENT

The corresponding author affirms that this manuscript is an honest, accurate, and transparent account of the study being reported; that no important aspects of the study have been omitted; and that any discrepancies from the study as planned (and, if relevant, registered) have been explained.

## Supporting information

Supporting Information.

Supporting Information.

Supporting Information.

## Data Availability

The data of the study are available by the corresponding author upon reasonable request.
