# Supplementary material for: Vitamin D prescribing practices among clinical practitioners during the COVID‐19 pandemic
Source: Health Sci Rep. 2022 Jul 11;5(4):e691. doi: 10.1002/hsr2.691 (PMC9273939; doi:10.1002/hsr2.691)
Supplement: Supplementary file 1 — Supporting Information. [file HSR2-5-0-s002.pptx]

## Slide 1
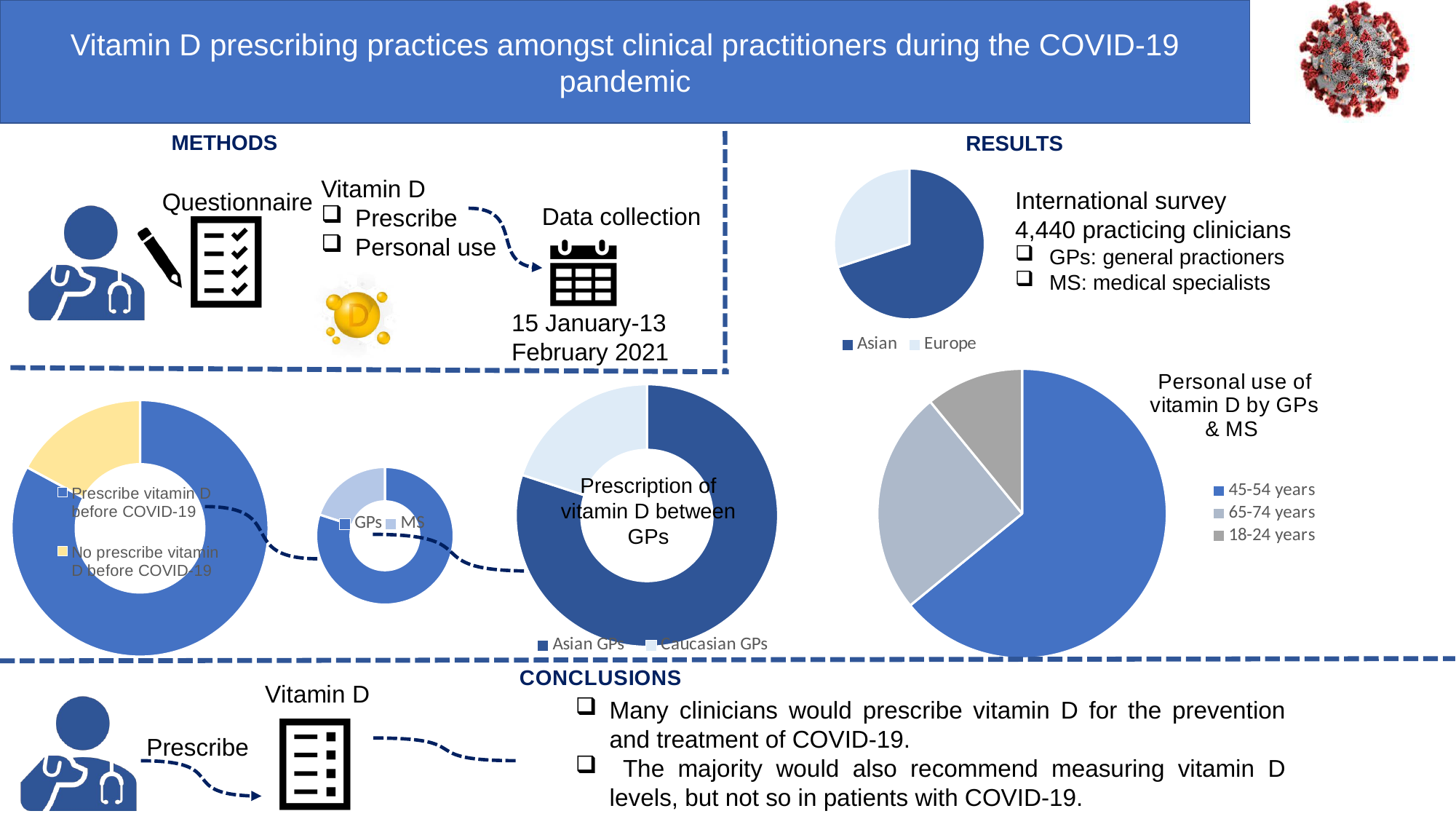

Vitamin D prescribing practices amongst clinical practitioners during the COVID-19 pandemic
METHODS
RESULTS
### Chart
| Category | Στήλη1 |
|---|---|
| Asian | 70.0 |
| Europe | 30.0 |Vitamin D
Prescribe
Personal use
International survey
4,440 practicing clinicians
GPs: general practioners
MS: medical specialists
Questionnaire
Data collection
### Chart
| Category | Πωλήσεις |
|---|---|
| Prescribe vitamin D before COVID-19 | 82.9 |
| No prescribe vitamin D before COVID-19 | 17.1 |
15 January-13 February 2021
[unsupported chart]
### Chart
| Category | Personal use of vitamin D |
|---|---|
| 45-54 years | 8.2 |
| 65-74 years | 3.2 |
| 18-24 years | 1.4 |
### Chart
| Category | Πωλήσεις |
|---|---|
| Asian GPs | 80.0 |
| Caucasian GPs | 20.0 |
### Chart
| Category | Στήλη1 |
|---|---|
| GPs | 80.0 |
| MS | 20.0 |Prescription of vitamin D between GPs
Μany clinicians would prescribe vitamin D for the prevention and treatment of COVID-19.
 The majority would also recommend measuring vitamin D levels, but not so in patients with COVID-19.
Prescribe
