# Supplementary material for: Vitamin D prescribing practices among clinical practitioners during the COVID‐19 pandemic
Source: Health Sci Rep. 2022 Jul 11;5(4):e691. doi: 10.1002/hsr2.691 (PMC9273939; doi:10.1002/hsr2.691)
Supplement: Supplementary file 2 — Supporting Information. [file HSR2-5-0-s001.docx]

**Supplementary Table 1.**

| **Location of respondent**, n (%)  Africa  Central and South America  Europe (other)  Greece  India  Middle East, Asia and Asia-Pacific (other)  North America  UK  Not stated | 16 (0.4)  217 (4.9)  288 (6.5)  218 (4.9)  2,999 (67.6)  55 (1.2)  217 (4.9)  430 (9.7)  6 (0.1) |
| --- | --- |
