# Supplementary material for: Vitamin D prescribing practices among clinical practitioners during the COVID‐19 pandemic
Source: Health Sci Rep. 2022 Jul 11;5(4):e691. doi: 10.1002/hsr2.691 (PMC9273939; doi:10.1002/hsr2.691)
Supplement: Supplementary file 3 — Supporting Information. [file HSR2-5-0-s003.docx]

**S1 file. Covid-19 survey amongst healthcare professionals:**

1. Are you a practicing physician?
   - 1. Yes. If YES please proceed to Q2
     2. No.
2. Speciality: Endocrinologist or Diabetologist / General Practitioner / Geriatrics / Gastroenterologist / Rheumatologist / Infectious Disease / Other (please state)
3. Ethnicity: Caucasian / South Asian / Black or African American / Chinese / Other (please state)
4. Age: 18-24 / 25-35 / 35-44 / 45-54 / 55-64 / 65-74 / Over 75
5. Which country do you work in? America / Canada / UK / France / Germany / Spain / Sweden / Italy / Australia / Russia / Other (please state)
6. Do you manage patients with Covid-19?
   - 1. Yes.
     2. No.
7. Do you treat patients with Covid-19? Y /N
   - 1. Outpatients.
8. In-patients (non-ICU).
9. ICU.
10. Community practice / GP surgery.
11. Did you prescribe Vitamin D before Covid-19?
    - 1. Yes.
      2. No.
12. Would you prescribe Vitamin-D to prevent Covid-19?
    - 1. Yes.
      2. No. (if No proceed to Q11)
13. If you would prescribe vitamin D to prevent Covid-19:

A. Only in patients who are vitamin D deficient

- - 1. Yes.
    2. No.

B. You would prescribe to everyone, irrespective of vitamin D status

- - 1. Yes.
    2. No.

1. Would prescribe vitamin D to TREAT Covid-19?
   - 1. Yes.
     2. No.
2. If you would prescribe vitamin D to treat Covid-19:

D. Only in patients who are vitamin D deficient

- - 1. Yes.
    2. No.

E. You would prescribe to everyone irrespective of vitamin D status

- - 1. Yes.
    2. No.

1. Do you take Vitamin D yourself?
   - 1. Yes.
     2. No.
     3. Prefer not to say.
2. If Yes to Q14:
   - 1. Before Covid.
     2. After Covid started.
3. If you are on vitamin D, were you Vitamin D deficient before commencing treatment?
   - 1. Yes.
     2. No.
     3. Don’t know.
     4. Prefer not to say.
4. If a family member had Covid-19, would you recommend vitamin D?
   1. Only if deficient in vitamin D levels.
   2. Irrespective of vitamin D levels.
   3. Would not recommend.
   4. Don’t know.
5. Should everyone be tested for vitamin D deficiency?
   1. Yes.
   2. Only if they have confirmed or suspected Covid-19.
   3. Only if they are at risk of deficiency.
   4. Only if they have confirmed or suspected Covid-19 OR if they are at risk of deficiency.
6. Patients with Covid-19 need not be tested for vitamin D deficiency.
   1. Agree.
   2. Disagree.
